# Embedded-structure template for electronic records affects patient note quality and management for emergency head injury patients: An observational pre and post comparison quality improvement study

**DOI:** 10.1097/MD.0000000000005105

**Published:** 2016-10-07

**Authors:** Tomohiro Sonoo, Satoshi Iwai, Ryota Inokuchi, Masataka Gunshin, Yoichi Kitsuta, Susumu Nakajima

**Affiliations:** The University of Tokyo Hospital Emergency Medicine and Critical Care Medicine Department, Hongo, Bunkyo-ku, Tokyo, Japan.

**Keywords:** checklist, clinical decision support, patient safety, quality of care

## Abstract

Along with article-based checklists, structured template recording systems have been reported as useful to create more accurate clinical recording, but their contributions to the improvement of the quality of patient care have been controversial. An emergency department (ED) must manage many patients in a short time. Therefore, such a template might be especially useful, but few ED-based studies have examined such systems.

A structured template produced according to widely used head injury guidelines was used by ED residents for head injury patients. The study was conducted by comparing each 6-month period before and after launching the system. The quality of the patient notes and factors recorded in the patient notes to support the head computed tomography (CT) performance were evaluated by medical students blinded to patient information.

The subject patients were 188 and 177 in respective periods. The numbers of patient notes categorized as “CT indication cannot be determined” were significantly lower in the postintervention term (18% → 9.0%), which represents the patient note quality improvement. No difference was found in the rates of CT performance or CT skip without clearly recorded CT indication in the patient notes.

The structured template functioned as a checklist to support residents in writing more appropriately recorded patient notes in the ED head injury patients. Such a template customized to each clinical condition can facilitate standardized patient management and can improve patient safety in the ED.

## 1 Introduction

Past studies have assessed article-based checklists to ascertain whether they contribute to improvement of clinical care, or not, yielding various results. Some reports suggest that such checklists can improve the safety of emergency procedures,^[1,2]^ but others have shown no reduction of surgical complications when used in presurgical settings.^[3]^ For emergency departments (EDs), only limited evidence supports checklist effectiveness. Some earlier studies have failed to provide evidence for the effectiveness of checklists for critically ill patient detection in pediatric EDs.^[4]^ Others have failed to demonstrate the quality and safety of ED handoffs.^[5]^

Compared to these article-based checklists, a structured reporting template emerged recently that can be input using computers connected to electronic health records (EHRs) and which can also be effective for use as checklists. These structured template systems have been generally accepted by medical staff members with high satisfaction.^[6]^ Additionally, they enable more clinically accurate recording than widely prevalent dictation-based writing systems.^[7–9]^ Furthermore, appropriately coded structured templates can be quite effective to collect data that are readily available for clinical studies.^[10]^

Studies specifically addressing such structured templates for specific clinical conditions have demonstrated that they contribute to quality improvement of information recording itself, but contributions to outcomes related directly to patient prognosis have remained controversial. Compliance to known guidelines or patient notes recording quality has been improved using structured templates for ankle injury^[11]^ or deep venous thrombosis,^[11]^ but neither study provided evidence for a change in patient outcomes. Regarding imaging study reporting, such structured templates have led to lower frequency of mistakes in findings,^[12]^ but no significant difference was found by one study.^[13]^ A structured template did achieve higher inhaled corticosteroid prescription in asthma patients^[14]^ and realized greater numbers of patients seen with shorter waiting times for rheumatoid arthritis patients.^[15]^

These results of several studies suggest that checklists and structured reporting templates can improve several indicators, but the contribution to clinical quality is difficult to assess.^[16,17]^ Few studies have demonstrated improved outcome measures that might be related directly to patient prognosis.^[12,14,15]^ Medical staff members generally expect that some means of data collection can improve clinical quality measures, but problems related to usability or accessibility of such systems might persist as obstacles to their wider use.^[18]^ In busy ED settings, to avoid unnecessary medical lawsuits and to realize patient safety, not only patient prognosis but also the quality of the patient notes per se might be important. These quality measures can be improved using an EHR-related structured template that is readily available in the ED.

We developed a structured reporting template based on widely used head injury guidelines, which are useful as an ED clinical management checklist. Then we analyzed the quality of patient notes and patient management.

## 2 Methods

**Figure 1:**
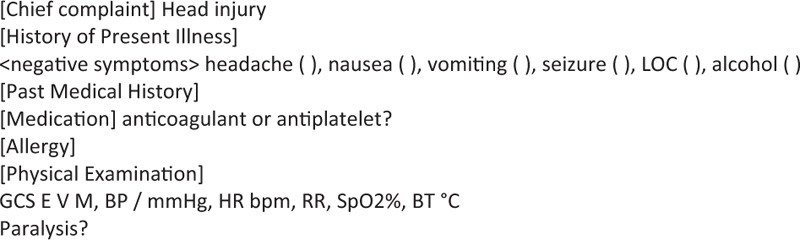
Structured template that was used. The template was in Japanese in actual clinical settings because the study was conducted in Japan.

The study was conducted at the University of Tokyo Hospital ED, which has 20,000 patients’ visit annually. In the ED, residents (post-graduate year 1 or 2) initially contact the patients and record the patient notes. Then they are checked and edited by senior residents (post graduate year 3–7). From July 2015, a structured reporting template had been launched (Fig. 1) for head injury patients. It is quite simple because it includes only the most important symptoms or findings that should be checked. The template was produced based on commonly used head injury computed tomography (CT) indications.^[19]^ It was programmed to extend automatically to the EHR when residents started to write the patient notes of the patients with the chief complaint of head injury.

This study compared data obtained during preintervention (July 1, 2014–December 31, 2014) and postintervention (July 1, 2015–December 31, 2015) periods. To avoid differences in the proficiency of residents, the pre and post periods were set with a 6-month interval. All patients aged 20 years or older who came to our ED with the chief complaint of head injury and who were seen by ED residents during the terms were included. Patients with an injury sustained >24 hours before and patients who came to our ED by transfer from another hospital were excluded.

Contents of the patient notes of these patients were blinded for terms and shuffled. The recorded date, the date information included in the patient note contents, and the specific text of the template were erased for blinding. Using the identical checklist format, blinded patient notes were evaluated by three 3rd-year medical students using the identical checklist format. They did not participate in the subjective patients’ care, and do not have sufficient knowledge about clinical medicine including the CT indication after head injury because clinical medicine curriculums start from 4th year in our medical school. Four categories of evaluation, “Presence,” “Absence,” “Not referred,” and “Not checked because of patient factors,” were used to evaluate the presence or absence of the loss of consciousness, seizure, nausea/vomiting, neurological deficit, drug or alcohol intake, coagulopathy including anti-coagulation or anti-platelet medications, signs of skull fracture, and injury above the clavicle. The category “not checked because of patient factors” was analyzed in the same way as “presence.”

After this, the data were unblinded. The patient age, level of consciousness on arrival, and CT performance and the CT results (confirmed by final radiologist imaging reports) were collected. Then the indication categories for head CT were judged in the 3 categories of “indication,” “no indication,” and “not determined,” according to the guidelines,^[19]^ combining the results of the patient note evaluations made by medical students and the patient levels of consciousness and age. Even if some points were not recorded or were categorized as “not referred,” the indication category was confirmed as long as the indication was judged by the clearly written information only. If the information was insufficient to judge the indication for the head CT, then the patient was categorized as “not determined.”

Patients in each period were compared in terms of CT performance, CT results, and the CT indication category based on the patient note information. CT performed for patients with CT indication categories of “no indication” or “not determined” were labeled as “unsupported CT performance.” The CT skip for patients with CT indication categories of “indication” or “not determined” were labeled as “unsupported CT skip.”

Patient severity was evaluated by the surrogate markers, the level of consciousness, rate of intracranial hemorrhage, and rate of admission among patients in respective periods. Then the quality of patient notes was evaluated by the proportion of patient notes with the CT indication categorized as “not determined.” In addition, the proportions of unsupported CT performance and unsupported CT skip were compared as surrogate markers of the patient management quality. These markers were compared between 2 periods using *χ*^2^ test of the differences in proportions. All statistical analyses were conducted using software (JMP Pro ver. 12; SAS Institute, Cary, NC.).

Retrospective data collection in our ED was approved comprehensively by the Ethics Committee of the University of Tokyo Department of Medicine. Furthermore, the study entailed no direct intervention to the patient.

## 3 Results

Total patients examined were 388. Of those, 23 patients were excluded by the predefined exclusion criteria. As a result, 365 patients were analyzed: 188 in pre-intervention period; 177 in post-intervention period. Table 1 presents patients’ baseline characteristics, signs and symptoms, CT performance and their results, disposition, and the patient note categories. Although not significant, patients in the postintervention period showed a tendency to be older, and to have a higher rate of admission. In addition, the rate of male sex, the rate of drug or alcohol intake, and the rate of the injury above the clavicle were significantly higher in the preintervention period. Table 2 presents the CT indication categories, CT performance, and their results for respective groups.

**Table 1:**
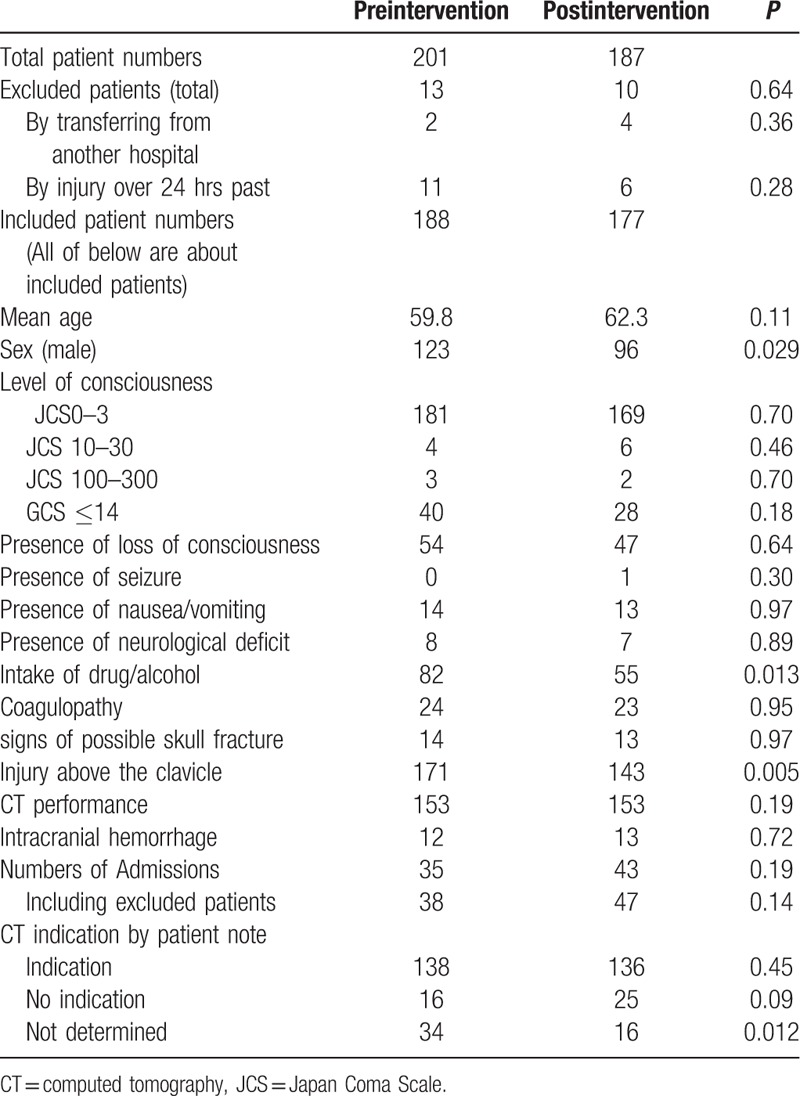
Detailed information related to patient numbers, characteristics, signs and symptoms, disposition, CT performance, and patient note quality.

**Table 2:**
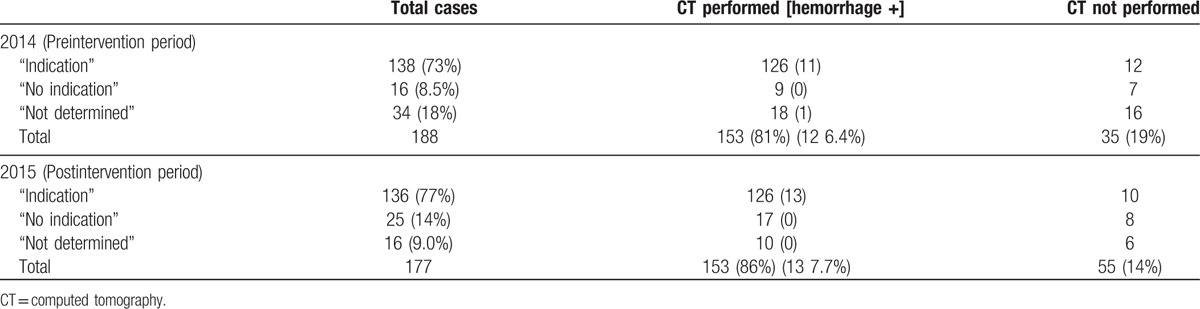
Numbers of cases, CT indication categories, and CT results for respective periods.

**Figure 2:**
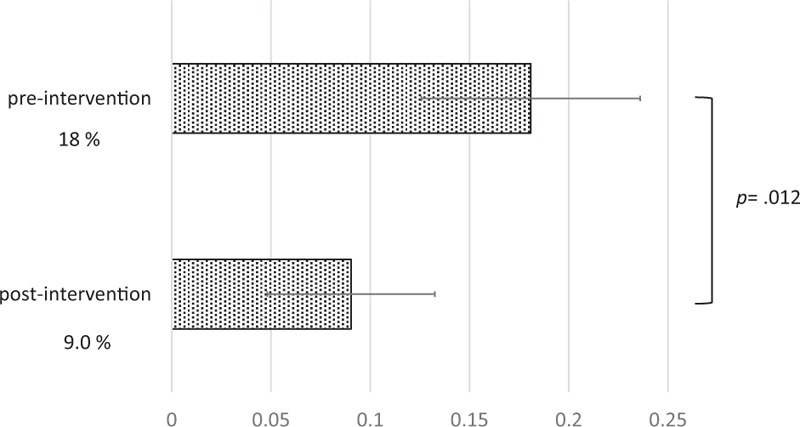
Proportions of patient notes categorized as “not determined” for the head CT indication in respective periods.

The Head CT performance rate and intracranial hemorrhage rate were not found to be significantly different between periods. Head CT was performed respectively in 153 of 188 cases (81%) and 153 of 177 cases (86%) in preintervention and postintervention periods (*P* = 0.19), respectively. Intracranical hemorrhage was found respectively in 12 of 188 (6.4%) and 13 of 177 (7.3%) in preintervention and postintervention periods (*P* = 0.72), respectively. Regarding the quality of patient notes, the proportions of patients with a CT indication category of “not determined” decreased significantly from 18% (34/188 patients) in the preintervention period to 9.0% (16/177 patients) in the postintervention period (*P* = 0.012). (Fig. 2) Although not significant, the proportion of patients with CT “no indication” category patients increased slightly from 8.5% (16/188 patients) to 14% (25/177 patients) in each period (*P* = 0.090).

**Figure 3:**
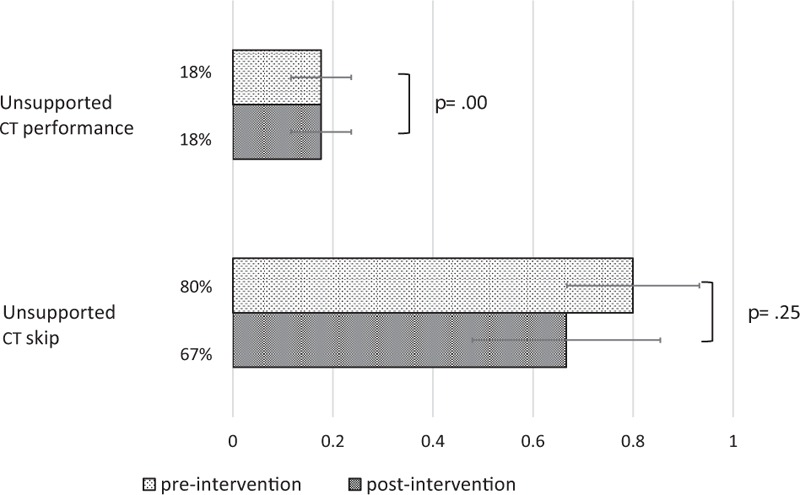
Proportions of the unsupported CT performance and unsupported CT skip in respective periods.

No difference was found between periods for the rates of unsupported CT performance and unsupported CT skip. Unsupported CT performance results were, respectively, 18% (27/153 patients) and 18% (27/153 patients) in preintervention and postintervention periods (*P* = 1.00). Furthermore, unsupported CT skip results were, respectively, 80% (28/35 patients) and 67% (16/24 patients) in preintervention and postintervention periods, respectively (*P* = 0.11) (Fig. 3).

## 4 Discussion

First, the patient severity and residents’ potential skill at writing the patient notes seem identical between the 2 periods. Although some differences exist in the baseline characteristics of subject patients between 2 periods, suggesting that the severity of patients would be slightly higher in the postintervention period, these differences were not significant. The rates of intracranial hemorrhage and patients’ level of consciousness were similar. In addition, the study terms were set with a 6-month interval, resulting in the same period in 1 year, which would certify that the residents’ skills and experiences at writing patient notes were expected to be roughly equivalent.

Using the structured template, the rates of patient notes categorized as the head CT indication of “not determined” have decreased significantly. This result demonstrates that the structured template functioned as a checklist for residents, which enabled us to elucidate the reasons underlying the CT performance when patient notes were checked retrospectively. This is particularly notable from the perspective of medical safety or to avoid unnecessary troubles between ED patients and medical staff members. In addition, about 1 of 5 patient notes in the preintervention term had been categorized as “not determined,” although these medical records were checked by senior residents or staff members, who were thought to have sufficient knowledge related to head CT indication. Results show that sole reliance on checking of manual patient notes is insufficient, but it might be efficient to use an appropriate template to compose records that include neither too much nor too little information. This kind of structured template would not only contribute to patient and resident safety. Residents would also learn fundamental knowledge related to emergency care in clinical conditions.

Reducing unnecessary CT performance or unnecessary CT skip might adversely affect patient outcomes in the ED. For that reason, unsupported CT performance and CT skip were set as outcome indicators. Results show no significant decrease of unsupported CT performances or unsupported CT skip in the postintervention period. However, the CT performance rate and admission rate were higher despite the tendency of increased proportions of “no indication” cases for head CT. This fact suggests that the reduction of “not determined” indication cases has engendered more thorough investigation of patients, resulting in appropriate CT performance and admission in high-risk patients.

This study specifically addressed head injury in the ED and revealed that the structured template can realize appropriate patient record construction in the ED and can facilitate standardized patient care according to the guidelines, patient safety, patient–physician conflict avoidance, resident education, and medical cost optimization by clarifying the indications of imaging studies. Future effect evaluation studies conducted with more sophisticated structured templates must be undertaken to examine other conditions that are frequently encountered in the ED. This approach has the potential to improve the quality of care for ED patients, not only in head injury patients but also in patients with other conditions that are known to be managed with clear guidelines and which are commonly encountered in the ED.

Finally, our study has several limitations. First, factors other than the launching of the structured template including the difference of numbers of cases, and difference of attending physicians or residents can affect the defined outcome markers. Second, the study is not a randomized control trial. The blinding we performed was not complete. Moreover, larger numbers of cases would probably have increased the meaningfulness of the difference of unsupported CT performance/skip results.

## 5 Conclusion

The use of a guideline-based structured template for patient notes has improved clinical record quality but has not changed the CT performance rate in the ED head injury patients. The template is expected to be useful in time-limited ED settings.

## Acknowledgments

The authors thank Wataru Mori, Shigemitsu Kojima, and Yasuyuki Tsushima from the University of Tokyo School of Medicine for the efforts of data analysis.
